# Altitudinal variation in haemosporidian parasite distribution in great tit populations

**DOI:** 10.1186/1756-3305-6-139

**Published:** 2013-05-07

**Authors:** Juan van Rooyen, Fabrice Lalubin, Olivier Glaizot, Philippe Christe

**Affiliations:** 1 Department of Ecology and Evolution, University of Lausanne, CH-1015 Lausanne, Switzerland; 2 Museum of Zoology, CH-1014 Lausanne, Switzerland

**Keywords:** Parasite prevalence, *Plasmodium*, *Haemoproteus*, *Leucocytozoon*, Lineages

## Abstract

**Background:**

One of the major issues concerning disease ecology and conservation is knowledge of the factors that influence the distribution of parasites and consequently disease outbreaks. This study aimed to investigate avian haemosporidian composition and the distribution of these parasites in three altitudinally separated great tit (*Parus major*) populations in western Switzerland over a three-year period. The objectives were to determine the lineage diversity of parasites occuring across the study populations and to investigate whether altitudinal gradients govern the distribution of haemosporidian parasites by lineage.

**Methods:**

In this study molecular approaches (PCR and sequencing) were used to detect avian blood parasites (*Plasmodium* sp., *Haemoproteus* sp. and *Leucocytozoon* sp.) in populations of adult great tits caught on their nests during three consecutive breeding seasons.

**Results:**

High levels of parasite prevalence (88-96%) were found across all of the study populations with no significant altitude effect. Altitude did, however, govern the distribution of parasites belonging to different genera, with *Plasmodium* parasites being more prevalent at lower altitudes, *Leucocytozoon* parasites more at high altitude and *Haemoproteus* parasite prevalence increasing with altitude. A total of 27 haemosporidian parasite lineages were recorded across all study sites, with diversity showing a positive correlation to altitude. Parasites belonging to lineage SGS1 (*P. relictum*) and PARUS4 and PARUS19 (*Leucocytozoon* sp.) dominated lower altitudes. SW2 (*P. polare*) was the second most prevalent lineage of parasite detected overall and these parasites were responsible for 68% of infections at intermediate altitude, but were only documented at this one study site.

**Conclusions:**

Avian haemosporidian parasites are not homogeneously distributed across host populations, but differ by altitude. This difference is most probably brought about by environmental factors influencing vector prevalence and distribution. The high occurrence of co-infection by different genera of parasites might have pronounced effects on host fitness and should consequently be investigated more rigorously.

## Background

A key aspect concerning host-parasite dynamics is the understanding of factors that determine and shape parasite community composition. Understanding the patterns and factors that govern how and where parasites occur may help determine which aspects shape disease transmission and persistence.

Avian haemosporidian parasites (*Plasmodium* sp., *Haemoproteus* sp. and *Leucocytozoon* sp.) have a worldwide distribution, and it is speculated that this wide distribution is due to a combination of high host mobility, migration, low parasite specificity and limited specificity in vector choice for hosts [1] (but see e.g. [2]), thereby enabling the transmission of parasites belonging to numerous genera of Haemosporida (*sensu*[1]). Currently, avian haemosporidian parasites are represented by over 130 species (that are distinguishable by morphological characters) of the genus *Haemoproteus*, over 50 species of the genus *Plasmodium* and 35 species of the genus *Leucocytozoon*[1,3]. With the development and refinement of molecular techniques for parasite identification, over 860 distinct parasite lineages (based on genetic variation located on the mitochondrial cytochrome *b* gene) have been described [4]. The genus *Haemoproteus* is divided into two subgenera - *H. (Parahaemoproteus)* and *H. (Haemoproteus)* - with the primary criteria for distinguishing the two being the vectors that transfer the parasites. In the case of *H. (Parahaemoproteus)*, the vectors include a variety of biting midges (Diptera: Ceratopogonidae), while *H. (Haemoproteus)* parasites are vectored by hippoboscid flies (Hippoboscidae) [1] and are currently known to occur only in the Columbiformes [1], Pelecaniformes [5] and Charadriiformes [6]. The present study refers to *H. (Parahaemoproteus)* whenever the term “*Haemoproteus*” is used. Blood sucking dipterans are also vectors of *Plasmodium* and *Leucocytozoon* parasites. Precise information on vectors for natural populations are scarce, but laboratory studies have shown female mosquitoes from the *Culex*, *Aedes* and *Culiseta* genera to be vectors of avian *Plasmodium* parasites [1] (although vector competence in laboratory studies does not necessarily mean it is a vector in the wild), with two recent papers [7,8] showing *Culex pipiens* to be potential vectors of avian malaria in Europe. Leucocytozoids, on the other hand, are transmitted by simuliid flies (Simuliidae) [1].

Using microscopy to detect infected red blood cells from bird blood smears, Van Riper *et al.*[9] investigated elevation as a determinant of avian haemosporidian prevalence and reported the highest *Plasmodium* parasite prevalence in Hawaiian island forest birds at elevations of around 1000-1300m in mesic and around 800-1250m in xeric habitats. Advances in the field of molecular based methods for parasite detection (i.e. PCR) have allowed for the identification of blood parasites at the lineage (haplotype) level and hence the characterisation of parasite communities [4]. As a result, a large number of studies have since focussed on tracking avian Haemosporida diversity and distribution [10-17], with particular emphasis on infection in migratory species [18-21]. To our knowledge, however, no study apart from that of [22], who characterised blood protozoan infection in 40 bird species over an elevational gradient in Australia, has looked at avian haemosporidian parasite prevalence, diversity and distribution in populations that differ altitudinally, yet situated close enough for bird movement to take place. This study aimed to depict this population structure in a common resident species, the great tit (*Parus major*), over an elevational gradient in Switzerland. Climate is closely linked to altitude. Therefore, vector-borne diseases could impact hosts differently at various elevations, as vector development and distribution could limit or facilitate parasite transmission.

The great tit is an excellent model species as they are abundant and readily nest in provided boxes. They are therefore easily caught and manipulated. Many studies have focussed on great tits and how haemosporidian parasites affect and interact with reproductive success [23-29]. Nevertheless, little is known about natural haemosporidian prevalences and the parasite lineages infecting these birds (however, see [30] and [7]). In this study, three altitudinally separated populations of great tits were sampled over a three year period and haemosporidian prevalence, diversity and distribution were assessed across time and space. This study aimed to contribute to the growing set of avian haemosporidian distribution and prevalence data.

## Methods

### Great tit sampling

Three study sites equipped with nestboxes situated along an altitudinal gradient in the Canton of Vaud, Switzerland were sampled during the great tit breeding season for three consecutive years, 2009-2011. The low altitude study site comprised three patches of forest located around the Dorigny campus of the University of Lausanne (46°31’N; 6°34’E; alt. 380m)(145 nests) on the banks of lake Léman and is classified as temperate broadleaf, mixed forests containing species such as European Beech (*Fagus sylvatica*). The mid-altitude forest of Monod (46°34’N; 6°24’E; alt. 668m) (114 nests) was situated between the towns of Ballens and L’Isle and is characterised by a mixture of large, dominant deciduous species of European Beech and European Alder (*Alnus glutinosa*). During spring, a large proportion of this study site floods, resulting in a transformation of the site into marshland. The higher altitude site (hereafter referred to as “high” altitude) was located in the forest spanning between the towns of Mont-la-Ville and La Praz (46°40’N; 6°20’E; alt.1000m) (52 nests) and consisted of primarily pure coniferous forests, comprised of European Silver Fir (*Abies alba*) and European Spruce (*Picea abies*). No direct climatic data were available for the three study sites. Climatic data were obtained by using available data from MeteoSwiss climate stations for the period between 1961-1990 for the other closest available locations, and were inferred from spatial climate mapping [31]. These data (average monthly temperature per year, monthly moisture index and the yearly sum of the mean monthly precipitation) are given in Table 1. Mosquito populations have been monitored for several years in the three study populations and potential *Plasmodium* parasite vectors, such as *Culex pipiens*[7], are frequent in the low altitudinal site, less frequent at the mid altitudinal site and have never been captured at high altitude, where only one mosquito species feeding on mammals (*Aedes* sp.) has been encountered. The distribution of other haemosporidian parasite vectors, i.e. biting midges or black flies, are poorly known in Switzerland in general, and these species have never been sampled at any of the current study sites. Nestboxes were inspected frequently and adult great tits were trapped therein by using door traps mounted inside the boxes when their nestlings were twelve days old. A 30 *μ*l blood sample was taken by brachial venapuncture, and collected in lithium-heparin lined Microvettes ^*â“‡*^ (CB 300 LH, Sarstedt, Germany). All birds were captured under license from the Swiss Federal Office for the Environment (number F044-0799), and in accordance with the Cantonal Veterinary Authorities of the Canton de Vaud, Switzerland (authorisation number 1730).

**Table 1:** Climatic data for the three study sites

**Location**	**T**_***ave***_	**M**_***ind***_	**Precipitation**
	**(° C)**	(**mm/month**)	(**mm/month**)
Low Altitude	9.6	5	971
Mid Altitude	8.2	22	1 161
High Altitude	5.0	83	1 696

Climatic data for the three study sites - low, mid and high altitude. “T _*ave*_” is average monthly temperature per year, “M _*ind*_” is the monthly moisture index, measured as monthly precipitation (P) minus monthly potential evapotranspiration (ETP), i.e. P - ETP and “Precipitation” indicates the yearly sum of the mean monthly precipitation.

### Molecular analyses

DNA extraction from blood was achieved using the DNeasy tissue extraction kit (QIAGEN) according to the manufacturer’s protocol for purification of DNA from blood, using the BioSprint 96. Subsequently, a nested PCR from the original protocol of [32], and refined by [33], was performed on all samples. The full method is described in [23] and [34].

Nested PCR products were purified using the Wizard SV Gel and PCR Clean-Up System (Promega) using the manufacturer’s protocol for DNA purification by centrifugation. Purified PCR products were sequenced as in [34] and identified by performing a local BLAST search with the MalAvi database [4].

Multiple double peaks observed on DNA chromatographs were confirmed to be indicators of multiple infections, i.e. concurrent infection with parasites from more than one lineage of the same genus. This was confirmed by cloning a randomly chosen subset of ten samples. This was done to verify that the observed double peaks indicated multiple infections by parasites within a single host, rather than amplification errors during sequencing. Cloning was performed using the pGEM ^*â“‡*^-T Easy Vector System (Promega) according to the manufacturer’s protocol. Ligation reactions were set up using purified PCR products and cultures of transformed high efficiency competent cells were plated onto LB/ampicillin/IPTG/X-Gal plates. Eight positive colonies were selected at random from every cloned sample. Inserts of positive colonies were sequenced using the SP6 and T7 Promoter Primers (Promega). See [34] for results. Co-infections were defined for this study as infections consisting of both *Leucocytozoon* and *Plasmodium* or *Haemoproteus* parasites concurrently identified within a host. Although molecular methods often underestimate multiple infections, especially between closely related parasites (see e.g. [35]), this was not the primary aim of this study. For the present purpose, the aim was to assess the identity of the parasites occurring within the study populations. However, slight possible over- or under-estimation of specific species detection (especially for *Plasmodium* and *Haemoproteus* in our populations) may occur with PCR-based detection compared to microscopical detection.

### Statistical analyses

All analyses were performed with the freeware R-Cran Project [36]. To determine whether there is a year or altitude effect on parasite prevalence, a linear mixed model (lmer) from the lme4 package [37] was used, with overall infection status as a binomial response variable. Year, altitude, as well as the interaction between these two variables were added as fixed effects and bird identity as a random factor to correct for recaptures. To determine whether the presence of parasites belonging to a certain genus was associated with altitude, a chi-squared test was performed. To assess the year effect on the prevalence of parasites belonging to different genera, likelihood ratio tests (LRT) were used to compare goodness of fit between models, similarly to the above mentioned model, but including genus-specific (*Plasmodium* and *Haemoproteus* prevalence and *Leucocytozoon* prevalence separately) infection status as response variable and excluding altitude as a fixed effect. Haemosporidian lineage richness and diversity were analysed using the vegan [38] and BiodiversityR [39] packages. The expected lineage richness for the entire surveyed area based on actual sampling effort and taking into account that lineages might not be randomly distributed across the sampling sites was determined with the Jacknife, Chao and Bootstrap formulae [39]. Rank-abundance curves were used to analyse patterns of lineage diversity using the logarithm of lineage abundance (abundance, in this special case, is defined as how prevalent a lineage is and not as the arithmetic mean of the number of individuals of a particular parasite species per host, examined as used in a classical parasitological textbook). The use of the logarithm of abundance when there are one or a few lineages that are highly dominant in a data set is recommended [39], as is the case with haemosporidian lineage SGS1 (*Plasmodium relictum*) [40]. Lineage diversity was further summarized by separately calculating the Shannon, inverse-Simpson and log series alpha diversity indices [39]. The Bray-Curtis distance calculation was used for lineage composition analysis between study sites as it copes better with different sample sizes between sites. As the data is dominated by parasites belonging to one lineage (SGS1), the data matrix was log transformed to correct for this skew in abundance. In the case of co-infections, Fisher’s exact tests were performed to determine whether altitude is a predictor of where co-infections between *Leucocytozoon* parasites and *Plasmodium* or *Haemoproteus* parasites occurred. Probability values of *p* ≤ 0.05 were considered significant.

## Results

### Great tit captures

A total of 328 adult great tits were captured during this study. In 2009, 110 great tits were captured (75 at low altitude, 27 at intermediate altitude and eight at high altitude), in 2010 a total of 158 (103 at low altitude, 40 at intermediate altitude and 15 at high altitude) and in 2011 a total of 60 great tits (43 at low altitude, 14 at intermediate altitude and three at high altitude) were sampled.

### Haemosporidian prevalence

**Figure 1:**
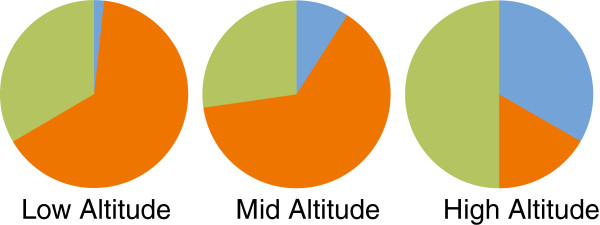
**Genera of avian haemosporidian parasites infecting great tits at three altitudes in western Switzerland.** Proportion (relative to each other) of avian haemosporidian parasites, by genus, recorded at a low, intermediate and high altitudinal study site in wild, breeding great tits (*Parus major*). Blue portions represent *Haemoproteus* sp. parasites, orange portions represent *Plasmodium* sp. parasites and green portions represent *Leucocytozoon* sp. parasites.

Avian Haemosporida parasite prevalence over three years was 96.4% (n = 110), 93.0% (n = 158) and 98.3% (n = 60) for 2009, 2010 and 2011 respectively. The high altitude population showed a parasite prevalence of 88.5% (n = 26) for all years combined, with low and intermediate altitudes showing 95.5% (n = 221) and 96.3% (n = 81), respectively, of birds being parasitized. No significant altitude or year effect was observed for this overall prevalence (GLM: *Z* = 0.39, n = 328, *p* = 0.695). However, when considering infection with *Plasmodium*, *Haemoproteus* and *Leucocytozoon* parasites separately, the above mentioned prevalence-by-altitude pattern changed (Table 2). Altitude was a significant determinant of the presence of parasites belonging to different genera (*χ*^2^ = 55.46, df = 4, n = 495, *p* ﹤ 0.0001), with *Plasmodium* parasites being more prevalent at low and intermediate altitudes than high altitude, *Leucocytozoon* parasites being more prevalent at high altitude than intermediate or low altitudes, and *Haemoproteus* parasites showing the highest prevalence at high altitude, less at intermediate altitude and the lowest at low altitude (Figure 1). There was no difference between years with respect to the number of individuals infected for neither *Plasmodium* nor *Haemoproteus* infections (LRT: *χ*^2^ = 3.16, df = 2, n = 328, *p* = 0.206), or *Leucocytozoon* ones (LRT: *χ*^2^ = 0.74, df = 2, n = 328, *p* = 0.691).

**Table 2:** Mean avian haemosporidian prevalences in great tits at three altitudes in Switzerland for three breeding seasons

	***Plasmodium***			***Haemoproteus***			***Leucocytozoon***			**Total**	
**Location**	**%**	***n***		**%**	***n***		**%**	***n***		**%**	***n***
Low Altitude	86.3	219		2.3	219		70.6	221		95.5	221
Mid Altitude	80.5	77		11.7	77		49.4	81		96.3	81
High Altitude	15.4	26		30.8	26		76.9	26		88.5	26
Total	79.2	322		6.8	322		65.9	328		95.1	328

Prevalence of avian haemosporidian parasite genera (*Plasmodium*, *Haemoproteus* &*Leucocytozoon*) in great tits (*Parus major*) at three altitudinally separate sites for three consecutive breeding seasons combined (recaptures are included). The “%” sign represents prevalence of birds infected with avian haemosporidians and “*n*” indicates the number of individuals sampled. For *Plasmodium* and *Haemoproteus* infections at low and mid altitude, the difference in birds sampled to that of total birds sampled is accounted for as the number of multiple infections that could not be assigned to either genus.

### Haemosporidian lineage diversity, richness and evenness

A combined total of 27 parasite lineages were recorded across all populations (four of them previously unencountered in the literature), including five *Plasmodium*, four *Haemoproteus* and 18 *Leucocytozoon* (Table 2). The low altitude study site showed a lineage richness of 17, the intermediate altitude a richness of 18 and the high altitude site a richness of 11. The total number of lineages expected to be present (based on actual sampling effort), when correcting for parts of the study areas not sampled, was predicted to be between 32 and 37 lineages in total (Jacknife=36.67, Chao=36.45, Bootstrap=32.22), of which we have sampled 27.

**Figure 2:**
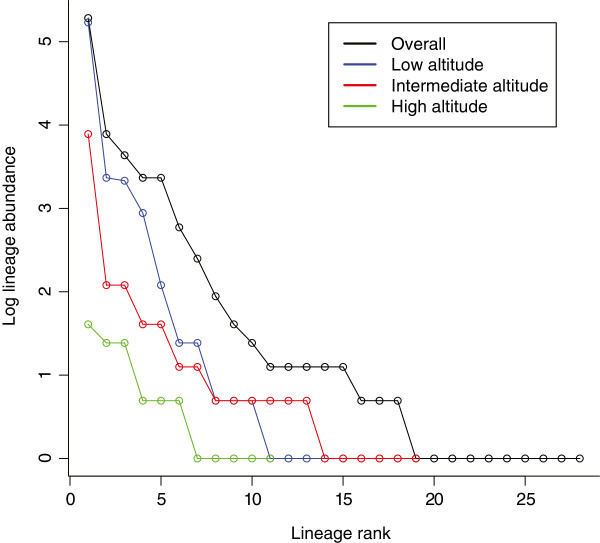
**Rank-abundance curve for avian haemosporidian lineages at three altitudes in western Switzerland.** Rank-abundance curve showing patterns of diversity for all avian haemosporidian parasite lineages found in three populations of great tits (*Parus major*) overall (black line), at low altitude (blue line), at intermediate altitude (red line) and at high altitude (green line). “Abundance” is defined as how prevalent parasites from each lineage are. The x-axis indicates lineage richness and the shape of the curve indicates evenness. The steeper the curve, the less evenly lineages are distributed (with a horizontal curve showing a completely evenly distributed system).

Rank-abundance matrices and curves showed high lineage richness and low evenness (Figure 2). All three diversity indices showed that high altitude has the highest diversity, intermediate altitude intermediate diversity and low altitude the lowest lineage diversity. According to the Bray-Curtis calculation used to determine differences in lineage composition between study sites, low and intermediate altitudes had the least amount of lineages in common, while low and high altitude had the most lineages in common (Bray-Curtis distances: low and mid altitude - 0.46; mid and high altitude - 0.56; low and high altitude - 0.64).

### *Plasmodium* and *Haemoproteus* parasite lineages

The most prevalent of all identifiable parasite lineages was SGS1 (*P. relictum*), with parasites belonging to this lineage infecting 95.9% (n = 195, number of infections which could be identified to lineage level for *Plasmodium* or *Haemoproteus*, see Table 2) of low altitude site birds, and 11.1% (n = 72) and 16.7% (n = 12) of the individuals at intermediate and high altitude, respectively, resulting in a total overall prevalence of 70.6% (n = 279). SW2 (*P. polare*) was the second most prevalent lineage, with these parasites being responsible for 17.6% (n = 279) of infections in great tits overall. However, SW2 parasites only occurred at the intermediate altitude site, where they infected 68.1% (n = 72) of the local population. PARUS1 (*H. majoris*) parasites were responsible for the majority of the *Haemoproteus* infections at 72.7% (n = 22, identifiable *Haemoproteus* lineages) (Table 3). All altitudes showed similar haemosporidian parasite lineage richness for *Plasmodium* and *Haemoproteus* parasites, with a total of five, six and five lineages detected at low, intermediate and high altitudes, respectively.

**Table 3:** Great tit haemosporidian lineage prevalence at three altitudes in western Switzerland for three breeding seasons

		**Low Alt. (380m)**			**Mid Alt. (668m)**			**High Alt. (1000m)**			
**Lineage**	**Morphospecies**	**2009**	**2010**	**2011**	**2009**	**2010**	**2011**	**2009**	**2010**	**2011**	**Total**
PARUS6	*Haemoproteus* sp.	–	1	–	–	–	–	–	–	–	1
PARUS1	*H. majoris*[41]	2	2	–	4	2	2	1	2	1	16
PHSIB1	*H. majoris*[41]	–	–	–	1	–	–	–	1	1(0)	3
WW2	*H. majoris*[41]	–	–	–	–	–	–	–	2	–	2
BT7	*Plasmodium* sp.	–	–	–	–	2	–	–	1	–	3
GRW11	*P. relictum*[42]	–	–	1	–	–	–	1	–	–	2
SGS1	*P. relictum*[40]	68	81(59)	38(21)	1	4	3(2)	1	1	–	197
SW2	*P. polare*[43]	–	–	–	16	27(23)	6	–	–	–	49
TURDUS1	*P. circumflexum*[40]	1	1	–	2	1	1	–	–	–	6
BT2	*Leucocytozoon* sp.	–	–	–	1	1	–	–	–	–	2
PARUS4	*Leucocytozoon* sp.	5	16	8(6)	1	3	1	1	3	–	38
PARUS16	*Leucocytozoon* sp.	1	3	4	1	1	–	1	–	–	11
PARUS18	*Leucocytozoon* sp.	1	3	–	2	1	–	–	–	–	7
PARUS19	*Leucocytozoon* sp.	9	15(11)	4(3)	–	1	–	–	–	–	29
PARUS20	*Leucocytozoon* sp.	1	–	1	1	2	–	–	–	–	5
PARUS22	*Leucocytozoon* sp.	11	8(6)	–	3	1(0)	1	3	2(1)	–	29
PARUS25	*Leucocytozoon* sp.	1	1	–	–	2	–	–	–	–	4
PARUS26	*Leucocytozoon* sp.	–	–	2	–	–	1	–	–	–	3
PARUS28	*Leucocytozoon* sp.	–	1	–	–	–	–	–	–	–	1
PARUS30*	*Leucocytozoon* sp.	1	–	–	–	–	–	–	–	–	1
PARUS31*	*Leucocytozoon* sp.	–	–	–	–	1	–	–	–	–	1
PARUS32*	*Leucocytozoon* sp.	–	–	–	–	1	–	–	–	–	1
PARUS33*	*Leucocytozoon* sp.	–	–	–	–	–	1	–	–	–	1
PARUS34	*Leucocytozoon* sp.	–	–	–	–	–	–	–	–	1	1
PARUS35	*Leucocytozoon* sp.	1	–	–	–	–	–	–	–	–	1
PARUS36	*Leucocytozoon* sp.	–	–	–	–	–	–	–	1	–	1
PARUS37	*Leucocytozoon* sp.	–	1	–	–	–	–	–	–	–	1
Total		102	133	58	33	50	16	8	13	3	416

Prevalence by altitude (Alt.) of haemosporidian parasite lineages in great tits (*Parus major*) for three breeding seasons. Citations (in square brackets) indicate references which assigned lineages to morphospecies. Parentheses denote new observations (i.e. not from a recaptured individual) when it was not the same as total observations. Multiple infections are excluded. *Lineages not previously encountered in the literature. GenBank accession numbers are given as an Additional file [see Additional 1].

### *Leucocytozoon* parasite lineages

**Figure 3:**
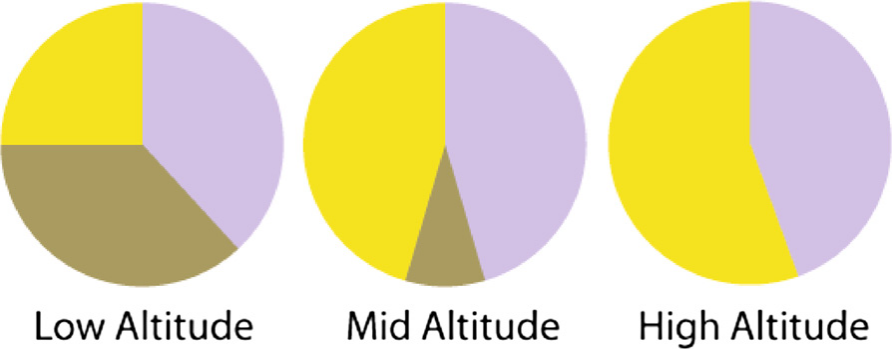
**Parasites from the main *Leucocytozoon * lineages infecting great tits at three altitudes in western Switzerland.** Proportion (relative to each other) of haemosporidian parasites belonging to the three most abundant *Leucocytozoon* lineages recorded at a low, intermediate and high altitudinal study site in wild, breeding great tits (*Parus major*). Purple portions represent parasites from lineage PARUS4, brown portions represent individuals from PARUS19 and yellow portions represent PARUS22 parasites.

The most prevalent *Leucocytozoon* parasite lineage detected was PARUS4, with individuals from this lineage responsible for 27.7% (n = 137 number of infections which could be identified to lineage level for *Leucocytozoon*, see Table 3) of all *Leucocytozoon* infections, and parasites belonging to lineages PARUS19 and PARUS22, respectively, responsible for 21.2% (n = 137) of infections. The distribution of parasites belonging to these three lineages across the three altitudes, however, differed (Figure 3). Although PARUS4 parasites declined in prevalence from low to intermediate altitude, they increased again at high altitude. PARUS22 parasites showed equal prevalences at low and intermediate altitude and increased in prevalence at high altitude. Apart from one isolated occurrence at intermediate altitude in 2010, PARUS19 parasites were only found at low altitude, even though it was one of the most prevalent lineages. Only parasites from five of the 18 detectable *Leucocytozoon* lineages were present at high altitude, whilst parasites from 12 out of the 18 were present at both low and high altitude (Table 3). The many occurrences of single detections of *Leucocytozoon* parasite lineages across years (Table 3) is most probably due to a combination of rare lineages being present and the lack of investigation into the *Leucocytozoon* parasites of passerines, with new lineages being discovered with each sampling.

### Multiple and co-infections

Multiple infections (i.e. concurrent infection with parasites belonging to more than one lineage of the same genus) in individual great tit hosts were found to occur at frequencies of 2.1% for *Plasmodium* and *Haemoproteus* infections (n = 285) and 33.8% for *Leucocytozoon* infections (n = 207, excluding positive infections that could not be scored as single lineages or multiple infections due to poor DNA chromatographs). Furthermore, 60.6% of all infected birds (n = 312) were co-infected (i.e. infections consisting of both *Leucocytozoon* parasites and *Plasmodium* or *Haemoproteus* parasites, or both, at the same time in a host) and these cases of multi-genus co-infections were most common at low altitude (*p* ﹤ 0.005, Fisher’s exact test).

## Discussion

A number of studies have investigated the interaction between avian haemosporidia infection and life-history traits in great tits [23-27,29], yet few attempts have been made to describe the occurrence and distribution of avian haemosporidia infection in this species. In fact, only one study by [24] has looked at haematozoa prevalence across time in a great tit population, but only recorded seasonal prevalence at the genus level. Studies by [12,44] and [17] have characterised prevalence and distribution of avian haemosporidian parasites in blue tits (*Cyanistes caeruleus*), and although being close relatives of great tits, such an effort has not been as extensive for the latter (but see [30] and [7]). Haemosporidian prevalences and parasite distribution in great tits recorded in this study were associated with altitude.

### Haemosporidian parasite prevalence and distribution

Although overall infection prevalences obtained in this study were comparable to previous studies on blue tits [17] and warblers (*Acrocephalus schoenobaenus*) [18], they were still notably higher than prevalences recorded in other passerines [11,15,20] and other areas. Analyses of environmental factors responsible for vector distribution and host susceptibility are needed to better understand haemosporidian distribution and abundance. Szöllősi *et al.*[17] found that, similarly to the current study, prevalence in blue tits differed with location, ranging from 30.5% to 100%, with an overall lineage richness of 13, six of which were recorded in the present study (BT7, GRW11, PARUS1, SGS1, SW2 and TURDUS1). [12] recorded an overall prevalence of *Plasmodium* and *Haemoproteus* parasites in blue tits of 28.4%, and an overall lineage richness of eleven (including four lineages recorded in the present study - BT7, GRW11, SGS1 and TURDUS1) of which, and similar to the results presented here, SGS1 (*Plasmodium relictum*, described by [45]) was by far the most prevalent lineage.

As far as we are aware, no previous study has characterised haemosporidian community composition in the same host species at different altitudes. The data suggest that there exists an altitudinal difference in the distribution of parasites belonging to different genera and different lineages within the same genus. While *Haemoproteus* parasite prevalence seems similar at all altitudes (Table 3), it appears that there are a lack of *Plasmodium* parasites at high altitudes. This is most probably due to a lack of mosquito vectors at higher altitudes [46]. *Haemoproteus* (*Parahaemoproteus*) parasites are vectored by biting midges (Diptera: Ceratopogonidae) [1]. Tschuor *et al.*[47] found that in Switzerland, biting midges are mostly linked to agriculturally utilised areas at higher altitudes. This could explain the slightly higher diversity and prevalence of *Haemoproteus* sp. found at intermediate and high altitudes in this study. With the exception of *Leucocytozoon (Akiba) culleryi*, which is transmitted by biting midges, all *Leucocytozoon* parasites are vectored by black flies (Diptera: Simuliidae) [1]. Imura *et al.*[48] reported high prevalence of *Leucocytozoon* parasites in willow tits (*Parus montanus*) and coal tits (*Parus ater*) at high altitudes in Japan (64.3% and 81.8% respectively), linking infection to black fly bites. The high prevalence of *Leucocytozoon* parasites observed at high altitude in this study could be due to the capacity of these parasites to successfully complete sporogony at relatively low temperatures [1], potentially as an adaptation to developing in high latitudes of the Holarctic (see [49] as an example of *L. simondi* in emperor geese, *Chen canagica*, in Alaska). The *Leucocytozoon* parasite prevalence at low altitude observed in the present study could be due to the fact that this study site borders a river on the north, as well as the site’s proximity to lake Léman (around 400 m) to the south. Simuliid flies have an excellent ability to colonise rivers or lakes [50], and can produce approximately a billion flies per day per kilometer of large river [51]. Information on Simuliid distribution in Switzerland and in our study sites in particular, however, is lacking.

The high prevalence of SGS1 parasites observed in this study (and elsewhere) was not unexpected, as *P. relictum* parasites are known to be generalists and wide-spread [19,40]. A more surprising finding was the high prevalence of SW2 parasites (*P. polare*) which, notably, only occurred at one site. Previous occurrences of SW2 (described in the sedge warbler, *A. schoenobaenus*[18]) have only ever been mentioned briefly [7,10,13,14,17,18] and very little information is available on these parasites. Vector distribution probably plays a role in the limited, and seemingly confined distribution of SW2 parasites. The intermediate altitudinal site serves as a breeding ground for a variety of mosquitoes, including *Culiseta* sp. and *Culex* sp., of which, [7] have recently shown *C. pipiens* to be potential vectors for these parasites. Even though very few *Culex pipiens* (also known as vectors for SGS1 parasites) are found at the intermediate altitude site in contrast to the low altitude study site where it is wide-spread, they are still the most abundant ornithophilic mosquito species at intermediate altitudes [7].

### Parasite community structure

The low prevalences with comparatively high lineage diversity found at intermediate altitude (especially in the case of *Leucocytozoon* infection) may be a result of stronger selective pressures imposed by exposure to a greater number of different parasites, which in turn, could result in adaptations that help evade or cope with actual infection [11]. The results indicate that this structure in parasite distribution for the three most abundant lineages (SGS1, SW2 and PARUS4) remains constant over time. It could be expected that local adaptation of hosts and parasites would take place in a spatially structured environment, as is the case for these three parasites. The interaction between these host-parasite rivals could differ between regions, as the ecological environment may influence them. This could then lead to a geographical mosaic of coevolution [52]. At the same time, the large number of blood parasite lineages that were found to occur in low numbers of hosts should impose on-going directional selection. However, an important consideration is that the high number of single-occurrences of lineages (PARUS6 and various *Leucocytozoon* lineages) could also be that of the “cul-de-sac” effect, i.e. parasites might be detectable in the blood of a host (due to circulating sporozoites or remnants of tissue meronts), but are unable to develop, resulting in a dead-end of the infection. Additionally, these parasites may not be able to complete sporogony in the vector to produce viable sporozoites - the essential stage for haemosporidian transmission, and as such are not viable and will not persist in the population.

### Multiple infections

The high rate of multiple infections (i.e. concurrent infection with multiple parasite lineages from the same genus) observed in great tits, especially for *Leucocytozoon* parasites, is not uncommon. Many studies have reported that parasites from several haemosporidian lineages can occur within one host species at any given time in a population [53-55]. Multiple infections from divergent parasite lineages could, however, result in the evolution of increased virulence because of intense competition for a limited number of hosts [56,57]. A study by Iezhova *et al.*[58] found that host susceptibility to different *Plasmodium* parasites differ in European passeriform birds. Palinauskas *et al.*[59] performed experimental multiple-infections and concluded that the virulence of such infections differed between host species and that it may even be absent in some. Studying different lineages from the same genus in distinct populations of avian hosts will lend fascinating insights into the complexities of host-parasite systems, with the emphasis on the dynamics of primary infection and interactions between different parasite lineages in multiple-infected hosts.

### Co-infections

Co-infections (i.e. concurrent infection with parasites from multiple genera) in avian malaria systems are more frequent than previously thought [53-55], with this study showing that over 60% of individuals could suffer infection from more than one genus of blood parasites at the same time. Although studies on co-infections in birds are rare, it has been shown that infection with two haemosporidian parasites in wild populations have much greater negative effects on house martins (*Delichon urbicum*) than single infections [60]. Furthermore, genetically different parasites may have contrasting physiological requirements, and as such, incur distinctly different sets of costs to their hosts. For example, Bentz *et al.*[54] showed that blackbirds (*Turdus merula*) experience different levels of parasitaemia when exposed to *Haemoproteus* parasites as compared to *Plasmodium* parasites. The effect of co-infection on host fitness and parasite virulence in great tits in this study system merits further investigation, especially considering the high co-infection rate observed.

### Great tits at high altitude

The small sample sizes obtained at the higher altitude could be attributed to great tit ecology. In Switzerland, great tits can be found at elevations of up to 1900m in the Alps, but is more of a lowland species, preferring mixed forest types and even being more partial to fragmented landscapes than dense pure deciduous forests, and actually disliking coniferous forest [61]. Coal tits (*Parus ater*) are adapted to life in coniferous forests (having long, non-opposable toes and claws, and fine bills), choosing almost exclusively to nest in this habitat, and showing particular preference to Spruce [61]. As a result hereof, great tits have been displaced by coal tits at the high altitude site, who occupy most of the nestboxes. Future studies should include recording haemosporidians in coal tits and blue tits that occur side-by-side with the great tits to see if they are infected with similar parasites and how this influences parasite communities.

## Conclusion

This study aimed to fill the gap between the massive amount of behavioural and life-history data available for great tits and the lack of information on the haemosporidians that inhabit them. Haemosporida parasites in these birds could have pronounced effects on their biology and behaviour, especially at such high prevalences as shown in the present study. Future work should focus in particular on the effects of co-infections on host populations.

## Competing interests

The authors declare that they have no competing interests.

## Authors’ contributions

PC and OG participated in the design of the study, assisted with data collection and manuscript drafting. FL contributed to data collection. JR collected data, performed molecular analyses, statistical analyses and drafted the manuscript. All authors read and approved the final manuscript.

## Supplementary Material

Additional file 1 **GenBank accession numbers for avian Haemosporida lineages.** GenBank accession numbers for all avian haemosporidian lineages sampled in three populations of great tits (*Parus major*) during this study. Click here for file
